# Magnetic resonance imaging‐based identification of the knee anterolateral ligament: A systematic review with meta‐analysis

**DOI:** 10.1002/jeo2.70816

**Published:** 2026-06-26

**Authors:** George Triantafyllou, Daniel Gondorf, Christos Koutserimpas, Nikolaos‐Achilleas Arkoudis, George Tsakotos, Maria Piagkou, Olympia Papakonstantinou

**Affiliations:** ^1^ Department of Anatomy, Faculty of Health Sciences, School of Medicine National and Kapodistrian University of Athens Athens Greece; ^2^ School of Health Rehabilitation Sciences University of Patras Patras Greece; ^3^ Second Department of Radiology General University Hospital “Attikon”, National and Kapodistrian University of Athens Athens Greece; ^4^ Research Unit of Radiology and Medical Imaging National and Kapodistrian University of Athens Athens Greece

**Keywords:** anterolateral corner, anterolateral ligament, knee, meta‐analysis, musculoskeletal radiology

## Abstract

**Purpose:**

The anterolateral ligament (ALL) is recognised as a stabiliser against internal tibial rotation and as a supporting structure for the anterior cruciate ligament; however, its MRI appearance is inconsistently reported. This systematic review and meta‐analysis aimed to synthesise the current literature to provide evidence regarding the visualisation, morphometry and injury prevalence of the ALL on magnetic resonance imaging (MRI).

**Methods:**

A systematic review of PubMed, Google Scholar, Scopus and Web of Science was conducted in accordance with preferred reporting items for systematic reviews and meta‐analyses guidelines. Eligible studies reported the MRI visualisation and/or anatomical characteristics of the ALL. Pooled prevalence and means were calculated using random‐effects models. Subgroup analysis and meta‐regression were performed to identify factors affecting visualisation.

**Results:**

Twenty‐six studies comprising 2706 knees were included. The pooled prevalence of ALL visualisation was 92.96% (95% CI: 87.81–96.91). Complete visualisation of the entire ligamentous course was achieved in 56.03% (95% CI: 37.39–73.86), while partial visualisation was 38.34% (95% CI: 27.90–49.32). Visibility was highest for the tibial part (80.91%) and lowest for the meniscal part (66.83%). Nationality (*p* < 0.001) and knee flexion (*p* = 0.0144) significantly influenced visualisation. Pooled morphometric means were length of 34.09 mm, width of 6.32 mm and thickness of 1.17 mm. Lastly, the pooled prevalence of concomitant ALL injury (with anterior cruciate ligament injury) was 35.63% (95% CI: 20.26–52.60).

**Conclusion:**

Pooled evidence suggests a high reported prevalence of ALL visualisation on MRI. However, considerable heterogeneity indicates that reproducibility across different protocols remains uncertain. While technical factors such as knee flexion and multiplanar assessment appear to enhance ALL identification, standardised radiological criteria are necessary to definitively distinguish the ALL from the adjacent joint capsule.

**Level of Evidence:**

Level III.

**Abbreviations**
ACL: anterior cruciate ligament
ALL: anterolateral ligament
ALLR: anterolateral ligament reconstruction
AQUA: anatomical quality assurance tool
CI: confidence interval
DOI: Doi Plot (diagnostic odds index plot, used for small‐study effects)
ITB: iliotibial band
LET: lateral extra‐articular tenodesis
LFK: Luis Furuya–Kanamori Index
MPR: multiplanar reconstruction
MRI: magnetic resonance imaging
PRISMA: preferred reporting items for systematic reviews and meta‐analyses

## INTRODUCTION

In recent years, the structures of the anterolateral knee have regained significant scientific attention following the detailed characterisation of the anterolateral ligament (ALL) through cadaveric and radiological studies [4, 21, 42]. Anatomically, the ALL typically originates from the prominence of the lateral femoral epicondyle. It follows an oblique course to insert on the proximal tibia, located between the Gerdy tubercle and the tip of the fibular head [4, 30]. Biomechanically, the ALL is considered to act as a stabiliser against internal tibial rotation and to influence the pivot‐shift phenomenon, particularly in anterior cruciate ligament (ACL) deficient knees [21]. Its clinical relevance is underscored by its close association with ACL injuries and avulsion fractures of the anterolateral tibia (Segond fracture) that are considered pathognomonic for ACL tears [21, 42].

Despite revived scientific interest, the morphology of the ALL remains controversial. While some cadaveric studies report its presence in nearly 100% of specimens, other investigations have found it to be absent in as many as 40% of cases [21, 31]. The recent meta‐analysis of cadaveric studies estimated the presence of ALL in 79% pooled prevalence [40]. Furthermore, some researchers suggest that the structure may not be a distinct ligament but rather a complex of fibrous tissues, including the fascia lata, fabellofibular ligament and intermuscular septum, that becomes taut during internal rotation [30].

**Figure 1:**
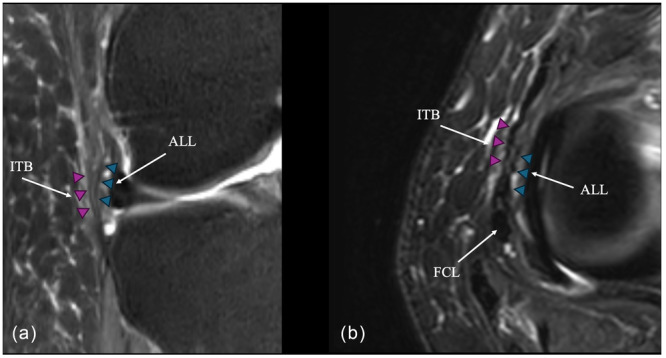
Coronal (a) and axial (b) reconstructions of T2‐weighted knee magnetic resonance imaging (MRI) scans of three Tesla. The anterolateral ligament (ALL‐blue arrows) is depicted, along with the iliotibial band (ITB‐purple arrows) and the fibular (lateral) collateral ligament (FCL).

Magnetic resonance imaging (MRI) is currently the most widely used imaging modality, although it is difficult to assess through clinical examination alone, particularly in the acute phase of injury (Figure 1). Identifying pathology in this region may be clinically relevant; however, there is no clear consensus that MRI‐detected anterolateral complex injuries, if unaddressed, independently result in chronic instability following ACL reconstruction.

A recent anatomical meta‐analysis of ALL did not include MRI studies [40]. Another systematic review depicted the MRI characteristics of the ALL [1]; however, there was no meta‐analytic synthesis. Therefore, the purpose of the current systematic review is to conduct an evidence‐based meta‐analysis of the MRI appearance of ALL by synthesising the current literature.

## MATERIALS AND METHODS

### Methodology

The current systematic review with meta‐analysis was performed in accordance with the preferred reporting items for systematic reviews and meta‐analyses (PRISMA) 2020 [32] guidelines. As the outcomes of this meta‐analysis were anatomical (anatomical visibility, anatomical parts, measurements), to detect possible publication bias, the anatomical quality assurance tool (AQUA) was used [18]. Five domains, each with questions and possible answers of ‘Yes, No or Unclear’, indicate the potential risk of bias as ‘Low, High or Unclear’. The protocol of this systematic review was registered in the PROSPERO database (CRD420261292311).

### Literature review synthesis

Two independent reviewers (G.T., D.G.) performed the literature search and data extraction. Results were compared, and the other authors settled potential differences. The terms ‘anterolateral ligament’, ‘anatomy’, ‘MRI’ and ‘magnetic resonance imaging’ were searched for in different combinations across the online databases PubMed, Google Scholar, Scopus and Web of Science through December 2025. MRI studies reporting the ALL anatomical characteristics and morphometry were selected as eligible to answer our research question. Exclusion criteria were case reports, conference abstracts and letters to the editor, as well as studies with irrelevant, insufficient or incomplete data; however, no language or data restrictions were imposed. Lastly, the references of all the studies included were examined for additional articles. The data were extracted into Microsoft Excel sheets before statistical analysis.

### Statistical analysis

Statistical analysis was conducted using the open‐source R programming language and RStudio version 4.3.2, with the ‘meta’ and ‘metafor’ packages, by a single researcher (G.T.). The pooled prevalence, along with its confidence interval (CI), was calculated using inverse variance and random‐effects models. The proportions meta‐analysis (prevalence meta‐analysis) was conducted using the Freeman‐Tukey double arcsine transformation, the DerSimonian‐Laird estimator for the between‐study variance *τ* ^2^, and the Jackson method for the CI of *τ* ^2^ and *τ*. The pooled mean analysis used untransformed values, the restricted maximum‐likelihood estimator for *τ* ^2^, and the Q‐profile method for CI. Cochran's *Q*‐statistic was used to evaluate the presence of heterogeneity across studies, and the Higgins *I* ^2^ statistic was used to quantify heterogeneity. Cochran's *Q p*‐value < 0.10 was considered significant. Higgins *I* ^2^ values between 0 and 40% were regarded as low heterogeneity, 30%–60% as moderate heterogeneity, 50%–90% as substantial heterogeneity and 75%–100% as considerable heterogeneity. A *p*‐value of less than 0.05 was considered statistically significant. To evaluate the robustness of the pooled prevalence estimates and their heterogeneity, a leave‐one‐out analysis was performed. To evaluate the presence of small‐study effects (the phenomenon in which smaller studies may show different effects than larger ones), the diagnostic odds index (DOI) (abbreviation from the author Suhail Doi) plot with the LFK (abbreviation from the author Luis Furuya‐Kanamori) index was generated [11]. Funnel plot asymmetry was tested using the Thomson‐Sharp method [35].

## RESULTS

### Study selection

**Figure 2:**
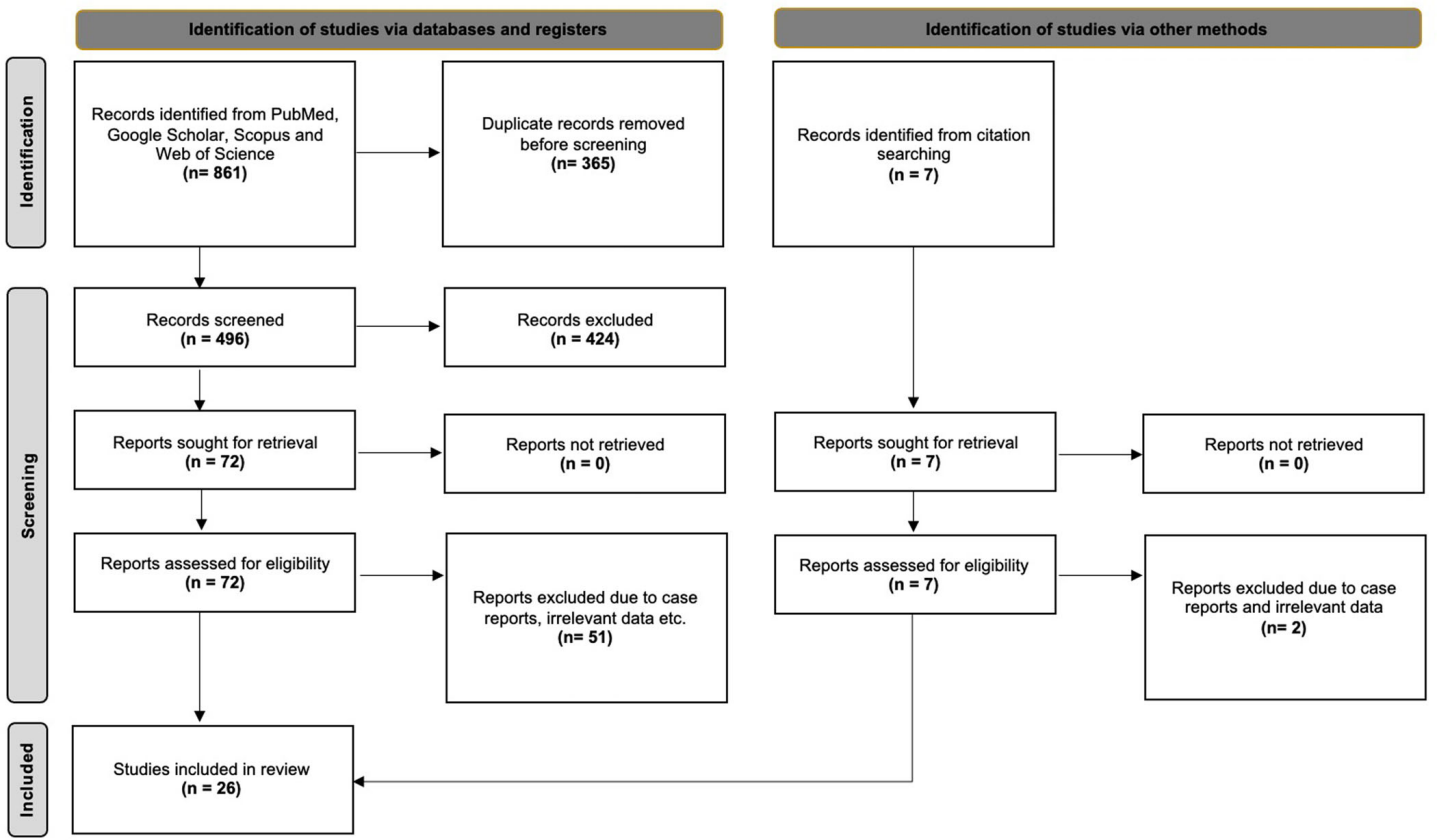
PRISMA 2020 flow chart diagram for literature search.

The initial database results yielded 861 articles, which were exported to Mendeley version 2.10.9 (Elsevier). After excluding irrelevant and duplicate papers, 72 studies were retrieved for full‐text screening. Finally, 21 studies were considered eligible for our initial meta‐analysis questions. Furthermore, 5 studies were identified from the reference check. Hence, 26 studies were included in the current systematic review with meta‐analysis. In accordance with the PRISMA 2020 guidelines [32]. Figure 2 presents the flow diagram of the selection process.

### Study characteristics

Twenty‐six studies were included, with a sample of 2706 knees. The mean sample per article was 84.56 knees. Twelve studies were conducted in the European population, eight in the North American and South American populations, five in the Asian population and only one in the Oceanian population. The methodological characteristics of each study (magnet, sequences, use of multiplanar reconstruction (MPR) and flexion of the knee) are summarised in Table 1.

**Table 1:** Characteristics of the included studies. Acquisition referred to the use of two‐dimensional (2D‐ single section) or three‐dimensional (3D)/multiplanar reconstruction (MPR). The type of sample referred to the inclusion of healthy or injured knees, with the most common cause being the anterior cruciate ligament injury.

Authors	Nationality	Magnet	Sequence	Slice (mm)	Acquisition	Flexion	Total sample	Type of sample	Risk of bias
Faruch Bilfield et al. [10]	Europe	1.5 T	T1	3.5	2D	10°	30	Injured	Low
Claes et al. [3]	Europe	Mixed	T2	Mixed	2D	‐	271	Injured	High
Coquart et al. [5]	Europe	1.5 T	T1	2	3D/MPR	15°	71	Mixed	Low
De Carli et al. [2]	Europe	1.5 T	Mixed	1.5	2D	None	26	Injured	High
Devitt et al. [7]	Oceania	3 T	T2	3	2D	None	127	Mixed	Low
Dimitriou et al. [8]	Asia	3 T	T1	1	2D	None	180	Mixed	Low
Hecker et al. [13]	Europe	3 T	T2	1	3D/MPR	None	94	Healthy	Low
Helito et al. [16]	South America	1.5 T	Mixed	Mixed	2D	15°	39	Healthy	Low
Helito et al. [14]	South America	1.5 T	Mixed	3	2D	‐	33	Healthy	Low
Helito et al. [15]	South America	1.5 T	T2	0.6‐1.5	2D	15°	13	Healthy	High
Helito et al. [17]	South America	1.5 T	T2	0.3	2D	‐	363	Healthy	Low
Huang et al. [19]	Asia	3 T	Mixed	0.5	3D/MPR	None	120	Healthy	Low
Kang et al. [20]	Asia	3 T	T2	3.5	3D/MPR	‐	66	Healthy	Low
Kızılgoz et al. [22]	Asia	1.5 T	Mixed	3.5	2D	15°	206	Injured	Low
Klontzas et al. [23]	Europe	Mixed	T1	0.4	3D/MPR	10°	46	Healthy	Low
Kosy et al. [24]	Europe	1.5 T	T1	3.5	2D	None	100	Injured	Low
Kosy et al. [25]	Europe	1.5 T	T1	3.5	2D	None	280	Injured	Low
Liebensteiner et al. [26]	Europe	Mixed	T1	3	2D	None	61	Healthy	Low
Macchi et al. [27]	Europe	1.5 T	Mixed	3.5	2D	‐	50	Healthy	Low
Marshall et al. [28]	North America	3 T	Mixed	‐	‐	30°	100	Mixed	Low
Muramatsu et al. [29]	Europe	3 T	T2	0.5	2D	30°	100	Mixed	Low
Porrino et al. [33]	North America	Mixed	Mixed	‐	2D	‐	73	Healthy	High
Song et al. [36]	Asia	3 T	T2	3	2D	‐	158	Injured	Low
Taneja et al. [39]	South America	Mixed	T1	3	2D	‐	70	Healthy	Low
Van Dyck et al. [9]	Europe	Mixed	Mixed	3	2D	‐	90	Injured	Low
Wodicka et al. [43]	North America	‐	‐	‐	‐	‐	50	Injured	High

The definitions of each study (ALL definition, complete visualisation, differentiation from the capsule and observers’ variability) are summarised in Table 2. The anatomical definition of the ALL was relatively consistent across the included literature, typically described as a low‐signal intensity linear or hypointense band originating from the lateral femoral epicondyle and inserting on the proximal tibia between Gerdy's tubercle and the fibular head. However, only 4 out of the 26 studies explicitly reported separating the ligament from the joint capsule, and an identical proportion (4/26) utilised the ‘triple‐layer’ sign to distinguish the ALL from the superficial iliotibial band (ITB) and the deep capsule. The definition of ‘complete visualisation’ varied, with most studies requiring the identification of all three anatomical portions (femoral, meniscal and tibial) to qualify. Studies were primarily performed by musculoskeletal radiologists (frequently working in pairs), though orthopaedic surgeons served as evaluators in four studies. Inter or intrarater reliability was generally high, with Cohen's kappa values ranging from 0.61 to 1.00 (Table 2).

**Table 2:** Characteristics of the included studies regarding the definitions and identification of the anterolateral ligament (ALL).

Authors	ALL definition	Capsule separation reported (Yes/No)	Complete ALL visualisation definition	‘Triple Layer’ sign	Evaluators specialty (number)	Reliability reported (value)
Faruch Bilfield et al. [10]	Low‐intensity linear structure between the femoral epicondyle and the tibial plateau	No	Visibility of each insertion separately (femoral, meniscal, tibial)	No	Radiologists (2)	Cohen's *k* (0.86–1.00)
Claes et al. [3]	Low‐signal‐intensity fibres arising from the lateral epicondyle, running obliquely to anterolateral border of the proximal tibia, passing lateral to the inferior geniculate vessels	No	NR	No	Orthopaedic surgeons (2)	NR
Coquart et al. [5]	Thin hypointense band from the lateral femoral epicondyle to the lateral tibial plateau between Gerdy's tubercle and fibular head	No	All three portions (femoral, meniscal, tibial) are seen, even if the insertion is not clearly individualised	No	Radiologists (2)	NR
De Carli et al. [2]	Low‐signal band from the lateral epicondyle, crossing the proximal surface of the LCL, deep to the ITB, inserting between Gerdy's tubercle and fibular head	No	NR	Yes	Radiologist (1)	NR
Devitt et al. [7]	Defined as separate from the capsule, LCL and ITB	Yes	Identified at ALL three sections (femoral, meniscal, tibial)	No	Radiologist (1) + Orthopaedic surgeon (1)	Cohen's *k* (0.83–0.88)
Dimitriou et al. [8]	Origin anterior and distal to LCL with ALL bifurcation into tibial and meniscal insertions, medial to lateral geniculate artery	No	NR	Yes	NR (2)	Cohen's *k* (0.87–0.99)
Hecker et al. [13]	Identified by anatomical landmarks (femoral insertion at lateral epicondyle, tibial midway between Gerdy's and fibular head)	No	Continuous ligamentous structure with both tibial and femoral insertion visible on a single coronal image	No	Radiologist (1) + Orthopaedic surgeon (1)	Cohen's *k* (0.61–0.88)
Helito et al. [16]	Identified between the LCL origin and the popliteus tendon proximally and inserting posterior to Gerdy's tubercle distally	No	All three portions visualised	No	Radiologists (2)	Cohen's *k* (0.84–1.00)
Helito et al. [14]	Similar to Helito et al. [16]	No	All three portions visualised	No	Radiologists (2)	NR
Helito et al. [15]	Similar to Helito et al. [16]	Yes	NR	No	Radiologist (2)	NR
Helito et al. [17]	Linear low‐signal structure in the anterolateral knee, originating near the LCL origin, anteroinferior path, attaching to the lateral meniscus and proximal tibia between Gerdy's tubercle and fibular head	No	NR	No	Radiologist (2)	Cohen's *k* (0.61)
Huang et al. [19]	Identified by its femoral footprint adjacent to the lateral epicondyle and LCL, and tibial footprint between the Gerdy tubercle and the fibular head	No	Clear footprints identified in all three proportions	No	Orthopaedic surgeons (2)	NR
Kang et al. [20]	Bone‐to‐bone ligament from the lateral femoral epicondyle to the lateral tibia	No	Bone‐to‐bone ligament from the lateral femoral epicondyle to the lateral tibia	No	Radiologist (2)	Cohen's *k* (0.66–0.82)
Kızılgoz et al. [22]	Thin ligament on the lateral side of the knee, surrounded by synovial fluid/adipose tissue, divided into femoral, meniscal and tibial parts	No	Entire ligament considered visible only when all three parts (femoral, meniscal, tibial)	No	Radiologist (2)	Cohen's *k* (0.78–1.00)
Klontzas et al. [23]	Origin at the lateral femoral epicondyle between the LCL and the popliteus, attachment to the meniscal body, distal insertion midway between Gerdy's tubercle and the fibular head	Yes	Confirmed by MPR showing common femoral footprint with LCL on oblique‐sagittal images, plus traceable course on coronal planes	No	Radiologist (2)	NR
Kosy et al. [24]	Ligament with femoral, meniscal and tibial portions; attachment to the lateral meniscus	No	All three portions (femoral + meniscal + tibial) visualised	No	Radiologist (1)	NR
Kosy et al. [25]	Similar to Kosy et al. [24]	No	NR	No	Radiologist (2)	Cohen's *k* (0.85)
Liebensteiner et al. [26]	Structure originating around the lateral femoral epicondyle, running anterodistally, inserting on the anterolateral tibia (passing the lateral inferior geniculate vessels)	No	NR	Yes	Radiologist (2)	Cohen's *k* (0.86)
Macchi et al. [27]	Thin linear structure originating at the lateral epicondyle (between LCL and popliteal tendon), running obliquely downward/forward, inserting on the lateral meniscus and the lateral proximal tibia	No	All three portions (femoral + meniscal + tibial) visualised	No	Radiologist (2)	NR
Marshall et al. [28]	Origin at the lateral epicondyle anterior to the LCL, insertion on the anterolateral tibia between Gerdy's tubercle and the fibular head	No	All three portions (femoral + meniscal + tibial) visualised	No	Radiologist (3)	Cohen's *k* (0.71)
Muramatsu et al. [29]	Low‐signal band originating from the region of the lateral femoral epicondyle, crossing the proximal surface of the LCL, and reaching the middle third of the lateral tibial plateau	Yes	NR	Yes	Orthopaedic surgeons (2)	Cohen's *k* (0.86 and 0.93)
Porrino et al. [33]	Similar to Claes et al. [3]	No	Described as visualised through entire course	No	Radiologist (2)	NR
Song et al. [36]	Divided into three anatomical parts on proton density‐weighted coronal images	No	NR	No	Radiologist (1) + Orthopaedic surgeon (1)	Cohen's *k* (0.81)
Taneja et al. [39]	Described as an intracapsular ligament with a close relationship with synovial tissue, ALL was only marked present when clearly seen on both axial and coronal sequences	No	All three thirds of its length are seen (superior, middle, inferior), even if a discrete meniscal/tibial/femoral attachment was not identified	No	Radiologist (2)	Cohen's *k* (0.70)
Van Dyck et al. [9]	Low signal intensity fibres from the lateral femoral epicondyle inserting into the anterolateral tibia	No	NR	No	Radiologist (2)	Cohen's *k* (0.74 and 0.79)
Wodicka et al. [43]	NR	No	NR	No	Radiologist (1)	NR

Abbreviations: ITB, iliotibial band; LCL, lateral collateral ligament; MPR, multiplanar reconstruction; NR, not reported.

### Primary meta‐analysis outcomes: ALL visibility

**Figure 3:**
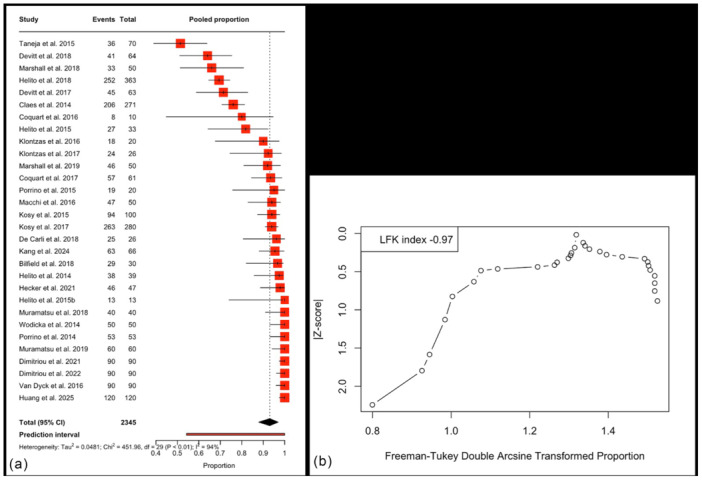
Forrest and DOI plots for the overall anterolateral ligament (ALL) visualisation pooled prevalence. DOI, diagnostic odds index.

The ALL visualisation was estimated using a pooled prevalence of 92.96% (95% CI: 87.81–96.91) (Figure 3). The *I* ^2^ was 93.6%, implying considerable heterogeneity. The leave‐one‐out analysis showed that the estimate was stable, with the shifted pooled prevalence ranging from 92.54% to 93.97% and the shifted *I* ^2^ ranging from 91.7% to 93.8% (Supplementary Material). The DOI plot had an LFK index of −0.97, indicating symmetry (Figure 3). Subgroup analysis of the ALL visibility is summarised in Table 3. Nationality and knee flexion subgroup analyses showed statistically significant differences (*p* < 0.001 and *p* = 0.0144, respectively). Lastly, a meta‐regression analysis was performed by slice thickness and revealed no statistically significant difference (*p* = 0.2421).

**Table 3:** Statistical meta‐analysis based on subgroup analysis for the overall visualisation of the anterolateral ligament (ALL).

Categories	Parameter	ALL visualisation (%)
Nationality (continents)	Europe (*n* = 12)	95.18
	Oceania (*n* = 1)	67.77
	Asia (*n* = 5)	99.60
	South America (*n* = 4)	82.18
	North America (*n* = 4)	94.13
	*p*‐value	<0.0001*
Magnetic strength (Tesla)	1.5 Tesla (*n* = 11)	92.04
	3 Tesla (*n* = 6)	94.98
	Mixed (*n* = 12)	87.12
	*p*‐value	0.5403
MRI sequence (T1‐/T2‐)	T1‐weighted (*n* = 9)	90.89
	T2‐weighted (*n* = 7)	98.52
	Mixed (*n* = 13)	89.22
	*p*‐value	0.2380
Technique for visualisation (2D/3D)	2D (*n* = 22)	92.85
	3D/MPR (*n* = 5)	95.81
	*p*‐value	0.5624
Knee flexion (No, 10°, 15°, 30°)	No Flexion (*n* = 9)	94.47
	10° Flexion (*n* = 3)	93.74
	15° Flexion (*n* = 4)	94.47
	30° Flexion (*n* = 2)	100
	*p‐*value	0.0144*
Patient status (healthy/injured)	Healthy (*n* = 18)	91.26
	Injured (*n* = 12)	95.01
	*p*‐value	0.3437

Abbreviations: 2D, two‐dimensional/single plane; 3D/MPR, three‐dimensional/multi‐planar reconstruction; MRI, magnetic resonance imaging.

* Depicting statistically significant *p*‐values.

**Figure 4:**
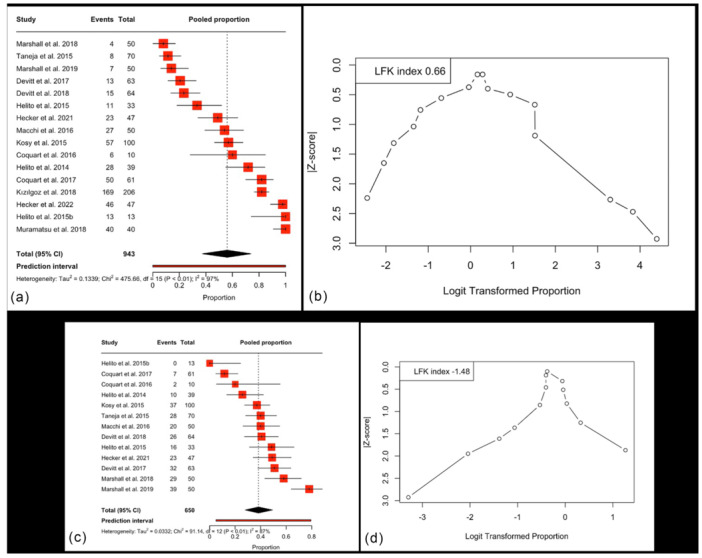
Forrest and DOI plots for the anterolateral ligament (ALL) complete visualisation pooled prevalence (a, b) and partial visualisation (c, d). DOI, diagnostic odds index.

Sixteen studies reported complete visualisation of ALL, and thirteen reported partial visualisation. Complete visualisation of the ALL was estimated at a pooled prevalence of 56.03% (95% CI: 37.39–73.86), while partial visualisation was estimated at 38.34% (95% CI: 27.90–49.32) (Figure 4). The *I* ^2^ were 96.8% and 86.8%, implying considerable and substantial heterogeneity, respectively. The leave‐one‐out analysis showed that the estimate for the complete visualisation was relatively stable, with the shifted pooled prevalence ranging from 51.58% to 59.43% and the shifted *I* ^2^ ranging from 96.4% to 97.1% (Supplementary Material). Similarly, for the partial visualisation, the shifted pooled prevalence ranged between 35.15% and 41.74%, and the shifted *I* ^2^ ranged between 81.3% and 87.9% (Supplementary Material). Complete visualisation DOI plot had an LFK index of +0.66 (no asymmetry), whereas the incomplete visualisation was −1.48 (minor asymmetry) (Figure 4).

### Secondary meta‐analysis outcomes: ALL anatomical parts

**Figure 5:**
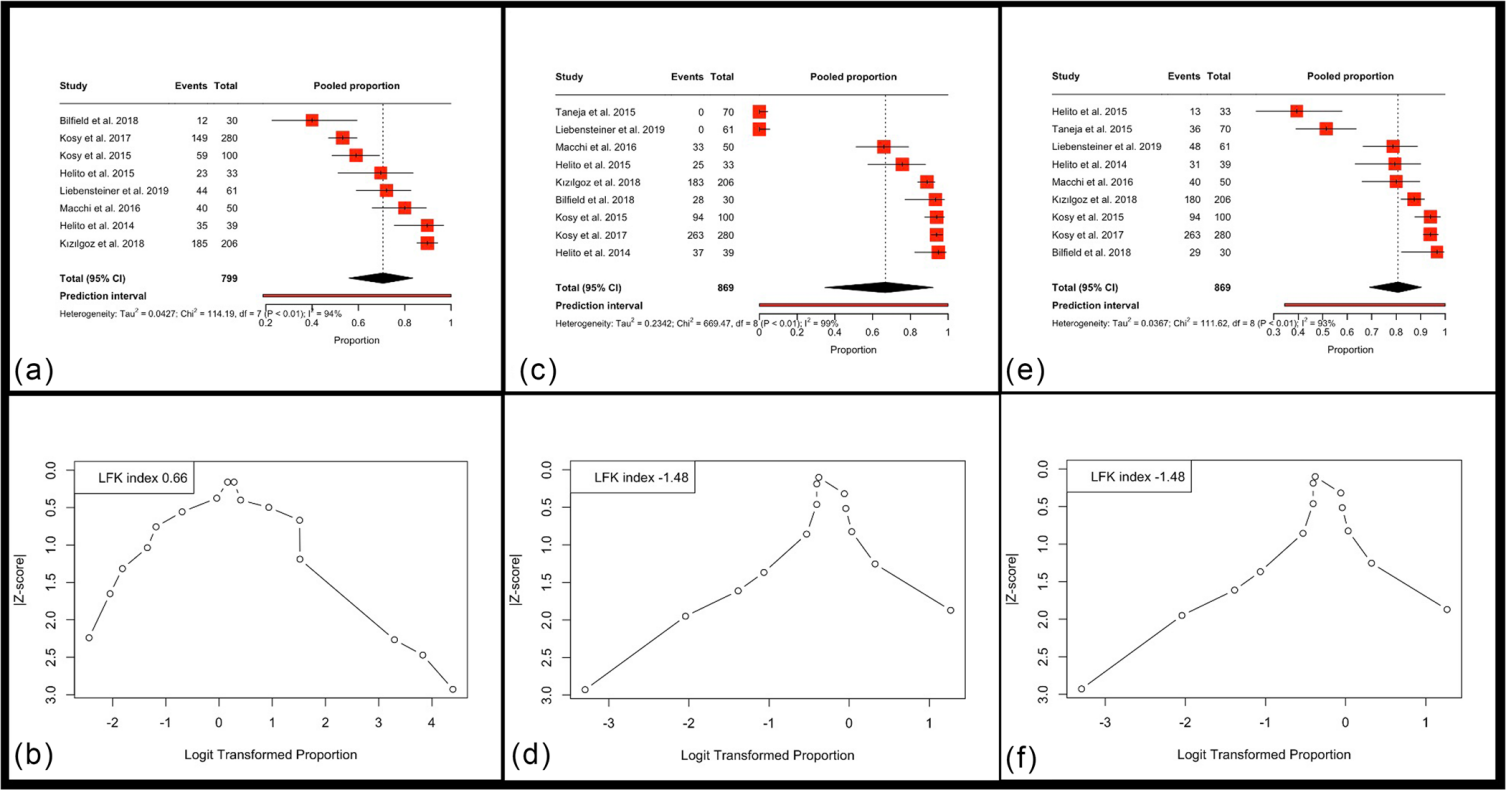
Forrest and DOI plots for the anterolateral ligament (ALL) visualisation of the femoral part (a, b), meniscal part (c, d) and tibial part (e, f).

Nine studies reported ALL visualisation based on its anatomical parts. The ALL‐femoral part (femoral origin) was visualised with a pooled prevalence of 70.78% (95% CI: 56.01–83.68) (Figure 5). The ALL‐meniscal part (meniscal attachment) was visualised with a pooled prevalence of 66.83% (95% CI: 34.74–92.27) (Figure 5). The ALL‐tibial part (tibial insertion) was visualised with a pooled prevalence of 80.91% (95% CI: 69.20–90.47) (Figure 5). The *I* ^2^ were 93.8%, 98.8% and 92.8%, implying substantial heterogeneity. The DOI plot of the ALL‐femoral parts was symmetrical, while the ALL meniscal and tibial parts were asymmetrical, indicating a possible small‐study effect (Figure 5).

### Secondary meta‐analysis outcomes: ALL measurements

Nine studies reported ALL morphometric characteristics. The ALL length was estimated to be 34.09 mm (95% CI: 32.31–35.87). The ALL width was calculated with a pooled mean of 6.32 mm (95% CI: 4.45–8.18). The mean ALL thickness was 1.17 mm (95% CI: 1.02–1.32). The Funnel plots’ asymmetry tests yielded *p*‐values of 0.982, 0.4674 and 0.0985, respectively, indicating symmetry. The leave‐one‐out analysis depicted that all prevalence estimates were relatively stable (Supplementary Material).

### Secondary meta‐analysis outcomes: ALL injury

An injured ALL was a concomitant finding (with ACL injury) with a pooled prevalence of 35.63% (95% CI: 20.26–52.60). The *I* ^2^ was 96.4%, implying considerable heterogeneity. The radiological characteristics (magnet, sequence used, MPR used and knee flexion angle) did not affect the estimated pooled prevalence of the injured ALL.

## DISCUSSION

The present systematic review and meta‐analysis represent the most comprehensive evaluation of the MRI appearance of the ALL to date, encompassing 26 studies and over 2700 knees. Our findings demonstrate a high pooled prevalence of ALL visualisation (92.96%), which, interestingly, exceeds the 79% presence reported in recent cadaveric meta‐analyses [40]. While this high visibility rate suggests the ALL is a consistent landmark, the high heterogeneity, its persistence after leave‐one‐out analysis, and our qualitative analysis (Table 2) reveal a significant lack of standardised diagnostic criteria. Only four studies explicitly differentiated ALL from the joint capsule. This suggests that the high pooled prevalence may reflect the identification of a ‘capsulo‐ligamentous complex’ rather than an independent ligament. Nevertheless, some statistically significant differences in technical parameters are particularly important findings because they can aid in the clinical identification of ALL injuries. Therefore, we will discuss them further.

### Optimisation of ALL identification

The high pooled prevalence of ALL visualisation found in this study (92.96%) suggests that ALL may be considered an important structure to assess during knee MRI interpretation. However, our finding that complete visualisation occurs in only 56.03% of cases highlights the technical challenges inherent in its identification. To maximise visualisation of the ALL, the radiologist should use isotropic 3D sequences that enable MPR, particularly in the coronal and axial planes. Our meta‐analysis demonstrated that knee flexion significantly influences visibility (*p* = 0.0144). In clinical practice, imaging the knee in slight flexion (10°–30°) tautens the anterolateral structures, reducing the wavy appearance often seen in full extension and making the ligament more distinct from the overlying ITB. As emphasised by Hecker et al. [13], the ALL rarely follows a single standard orthogonal plane. Therefore, the use of MPR by orienting the reconstruction axis along the oblique course from the lateral femoral epicondyle to the region posterior to Gerdy's tubercle is recommended [13]. With correct orientation, the femoral origin (proximal and posterior to the lateral epicondyle, slightly superficial to the fibular collateral ligament origin), the meniscal attachment (thin fibrous connection to the periphery of the lateral meniscus body), and the tibial insertion (approximately midway between the Gerdy tubercle and the fibular head) should be depicted [4, 13, 30]. According to the current meta‐analysis, the meniscal attachment is the most difficult to observe, with a pooled prevalence of 66.8%.

With a pooled injury prevalence of 35.63%, identifying ALL injury is a key component of the knee MRI report. Following the classification suggested by Muramatsu et al. [29] and confirmed by the findings of Kosy et al. [25], it is recommended to look for three specific radiological signs: (i) signal abnormality‐ increased T2 (with fat suppression) signal intensity within or surrounding the ligamentous fibres, (ii) morphological alteration‐ Frank discontinuity, thinning or an irregular contour suggests a loss of tension and (iii) avulsion (Segond fracture)‐ the radiographic ‘pathognomonic’ equivalent of a tibial ALL avulsion [9]. Even in the absence of a visible bone fragment, localised edema at the tibial insertion site should be reported as a Segond‐type injury [25].

The primary challenge remains differentiating ALL from the ITB. Devitt et al. [7] expressed scepticism regarding MRI reliability because these layers are closely apposed. To avoid false positives, the radiologist should look for the ‘triple‐layer’ appearance on axial slices: the superficial ITB, the intermediate ALL, and the deep capsule [7]. This sign was evaluated in only four studies in the current meta‐analysis. Its universal use would increase the correct identification of the ALL with a standardised protocol and reduce heterogeneity across studies.

### Technical parameters of ALL identification

One of the most significant findings is the high rate of partial visualisation (38.34%) compared to complete visualisation (56.03%). From a radiological perspective, this is likely due to the ligament's thin, ribbon‐like morphology and its oblique course across the joint line, which often requires multiple slices or MPR for a full assessment [13]. Hecker et al. [13] demonstrated that MPR significantly improves identification, which may explain the more consistent visualisation reported in studies utilising 3D MRI reconstructions, such as Muramatsu et al. [29].

Interestingly, although our meta‐regression did not find slice thickness to be statistically significant, the considerable heterogeneity in our results suggests that magnet strength and specific sequences remain influential. Marshall et al. [28] emphasised that while 3 Tesla (T) MRI provides high‐resolution images, interrater agreement for identifying the ALL remains only ‘fair to moderate’.

Our meta‐analysis identified nationality as a significant factor in ALL visibility. This is supported by recent 3D MRI analyses, such as those of Huang et al. [19], who documented race‐ and sex‐based variations in the ALL footprint. Such morphological diversity may explain the ALL morphology observed in the literature, ranging from distinct ligamentous bands to more diffuse capsular thickenings [30]. Furthermore, our results should be interpreted with caution in younger populations. Helito et al. [17] noted a lower visualisation rate (69.4%) in paediatric and adolescent patients, suggesting that the ALL may undergo progressive collagen condensation or maturation that increases its MRI conspicuity in adulthood.

Another important factor to consider is the expertise of the MRI evaluators. Our qualitative analysis (Table 2) revealed a lack of standardised diagnostic criteria across studies. Although most investigations were performed by experienced musculoskeletal radiologists and generally demonstrated high inter and intrarater reliability, reproducibility was not universal. Cohen's kappa values as low as 0.61 in some cohorts suggest that identification remains subjective and highly dependent on the observer's familiarity with the complex multi‐layered anatomy (Table 2).

### Clinical relevance of ALL recognition and surgical management

Recognition of ALL injury on MRI has direct clinical implications in the setting of ACL rupture, as untreated anterolateral insufficiency has been associated with persistent rotatory instability and a higher risk of residual pivot shift following isolated ACL reconstruction, as well as higher re‐rupture of the ACL [6, 12, 37, 41]. With a pooled prevalence of approximately one‐third of ACL‐injured knees demonstrating concomitant ALL injury, systematic assessment of the anterolateral structures should form part of routine preoperative planning. The presence of concomitant injury (ACL tear) may paradoxically aid ALL visualisation. The resulting hemarthrosis can act as a natural contrast agent, outlining the anterolateral structures. This may explain why studies with injured patients reported higher visualisation rates compared to those involving healthy knees, where the lack of joint effusion makes thin fibrous structures harder to delineate; even though this subgroup analysis was not statistically significant (Table 3).

From a surgical perspective, two main strategies have been adopted to address this pathology: anatomic ALL reconstruction (ALLR) and lateral extra‐articular tenodesis (LET) [34, 38]. Both aim to restore control of internal tibial rotation and reduce pivot shift, but through different biomechanical principles, with ALLR attempting to reproduce the native ligament course, while LET provides a nonanatomic check‐rein using the ITB [34].

Adjunctive anterolateral procedures are most commonly indicated in high‐risk situations, including high‐grade pivot shift, revision ACL reconstruction, young and highly active patients, generalised ligamentous laxity, chronic ACL deficiency, Segond fractures and confirmed ALL injury on imaging [34]. Growing clinical evidence suggests that combining ACL reconstruction with either ALLR or LET can reduce residual rotational laxity and lower graft failure rates in selected populations. Therefore, accurate MRI identification of ALL integrity is not merely descriptive but plays a key role in risk stratification, surgical decision‐making and individualised treatment planning in ACL‐injured patients.

### Strengths and limitations

The primary strength of this study is that it represents the first evidence‐based meta‐analysis specifically dedicated to the MRI appearance and morphometry of ALL. By synthesising data from 26 studies and 2706 knees, we have provided statistical power that individual investigations could not achieve alone. Unlike previous reviews that were purely qualitative [1], our use of subgroup analysis and meta‐regression allowed us to identify knee flexion and nationality as moderators of visualisation, providing practical guidance for optimising imaging protocols.

Despite these strengths, several limitations must be acknowledged. First, a diagnostic accuracy meta‐analysis was not possible for ALL identification because MRI findings were not directly comparable with cadaveric or surgical reference standards. Therefore, the true sensitivity and specificity of MRI for detecting ALL and accurately defining its anatomy remain unknown. In the context of MRI‐anatomical studies, a high risk of bias within the AQUA framework primarily reflects inconsistent anatomical definitions, variability in imaging acquisition protocols, limited reporting of observer reliability or blinding, and uncertainty regarding differentiation of the ALL from adjacent capsular or ITB structures. Second, we observed considerable heterogeneity across several primary outcomes, which persisted after the leave‐one‐out analysis. Although such heterogeneity is common in anatomical meta‐analyses due to interindividual variation, it also likely reflects the absence of a standardised radiological definition of ALL. Variability in ALL definitions, together with differences in intra and interobserver reliability, is summarised in Table 2. In addition, the blinding status of MRI evaluators was inconsistently reported across studies. Thirdly, the included studies utilised a wide variety of MRI protocols, ranging from 1.5 T to 3 T magnets and differing imaging sequences, which may have further contributed to heterogeneity. Lastly, our subgroup analysis suggested that nationality significantly influenced ALL visibility, implying that these findings may not be fully generalisable across all ethnic populations without further investigation.

## CONCLUSIONS

This systematic review and meta‐analysis demonstrate that a structure consistent with the anatomical description of the ALL is frequently reported in the MRI literature. However, the high statistical heterogeneity and inconsistent reporting of technical variables, such as the distinction between the ligament and the joint capsule, suggest that a universal identification protocol has not yet been established. Although optimisation strategies such as MPR and slight knee flexion appear to improve visualisation of the anterolateral corner, their diagnostic accuracy has not yet been validated across diverse populations. Future research should focus on standardising the radiological protocol and investigating the ALL appearance across ethnicities to further refine its clinical utility.

## AUTHOR CONTRIBUTIONS

**George Triantafyllou**: Conceptualisation; project development; data collection; statistical analysis; manuscript writing. **Daniel Gondorf**: Data collection; data analysis; manuscript writing. **Christos Koutserimpas**: Data analysis; manuscript review and editing. **Nikolaos‐Achilleas Arkoudis**: Data analysis, manuscript review and editing. **George Tsakotos:** Supervision; data analysis; manuscript review and editing. **Maria Piagkou**: Supervision; data analysis; manuscript review and editing. **Olympia Papakonstantinou**: Supervision, data collection; manuscript review and editing. All authors have read and approved the final version of the article.

## FUNDING

The authors have no funding to report.

## CONFLICT OF INTEREST STATEMENT

Christos Koutserimpas is a consultant for Smith and Nephew and has received the ‘MEDIKUS’ funding programme from the University of Patras, Greece. The remaining authors declare no conflicts of interest.

## ETHICS STATEMENT

The authors have nothing to report.

## Supporting information

Supporting information file 1.

## Data Availability

Please contact the authors for data requests.

## References

[1] Ariel de Lima D , Helito CP , Lacerda de Lima L , de Castro Silva D , Costa Cavalcante ML , Dias Leite JA . Anatomy of the anterolateral ligament of the knee: a systematic review. Arthroscopy. 2019;35(2):670–681. 30612770 10.1016/j.arthro.2018.09.006

[2] De Carli A , Monaco E , Mazza D , Argento G , Redler A , Proietti L , et al. Assessment of the anterolateral ligament of the knee by magnetic resonance imaging. Joints. 2018;6(3):153–156. 30582102 10.1055/s-0038-1675163 PMC6301852

[3] Claes S , Bartholomeeusen S , Bellemans J . High prevalence of anterolateral ligament abnormalities in magnetic resonance images of anterior cruciate ligament‐injured knees. Acta Orthop Belg. 2014;80(1):45–49. 24873084

[4] Claes S , Vereecke E , Maes M , Victor J , Verdonk P , Bellemans J . Anatomy of the anterolateral ligament of the knee. J Anat. 2013;223(4):321–328. 23906341 10.1111/joa.12087 PMC3791125

[5] Coquart B , Le Corroller T , Laurent PE , Ollivier M , Pradel V , Champsaur P , et al. Anterolateral ligament of the knee: myth or reality? Surg Radiol Anat. 2016;38(8):955–962. 26935828 10.1007/s00276-016-1657-2

[6] D'Ambrosi R , Carrozzo A , Monaco E , Sconfienza LM , Herbst E , Herbort M , et al. Lateral extra‐articular procedures reduce the risk of revision of anterior cruciate ligament reconstruction in elite athletes: a systematic review and meta‐analysis of comparative studies. Am J Sports Med. 2026;54(2):457–464. 41508654 10.1177/03635465251376655 PMC12861538

[7] Devitt BM , O'Sullivan R , Feller JA , Lash N , Porter TJ , Webster KE , et al. MRI is not reliable in diagnosing of concomitant anterolateral ligament and anterior cruciate ligament injuries of the knee. Knee Surg Sports Traumatol Arthrosc. 2017;25(4):1345–1351. 28405740 10.1007/s00167-017-4538-2

[8] Dimitriou D , Zou D , Wang Z , Helmy N , Tsai T‐Y . 3T MRI‐based anatomy of the anterolateral knee ligament in patients with and without an ACL‐rupture: Implications for anatomical anterolateral ligament reconstruction. Knee. 2021;29:390–398. 33706030 10.1016/j.knee.2021.02.007

[9] Van Dyck P , Clockaerts S , Vanhoenacker FM , Lambrecht V , Wouters K , De Smet E , et al. Anterolateral ligament abnormalities in patients with acute anterior cruciate ligament rupture are associated with lateral meniscal and osseous injuries. Eur Radiol. 2016;26(10):3383–3391. 26747257 10.1007/s00330-015-4171-8

[10] Faruch Bilfeld M , Cavaignac E , Wytrykowski K , Constans O , Lapègue F , Chiavassa Gandois H , et al. Anterolateral ligament injuries in knees with an anterior cruciate ligament tear: contribution of ultrasonography and MRI. Eur Radiol. 2018;28(1):58–65. 28702800 10.1007/s00330-017-4955-0

[11] Furuya‐Kanamori L , Barendregt JJ , Doi SAR . A new improved graphical and quantitative method for detecting bias in meta‐analysis. Int J Evid Based Healthc. 2018;16(4):195–203. 29621038 10.1097/XEB.0000000000000141

[12] Getgood AMJ , Bryant DM , Litchfield R , Heard M , McCormack RG , Rezansoff A , et al. Lateral extra‐articular tenodesis reduces failure of hamstring tendon autograft anterior cruciate ligament reconstruction: 2‐year outcomes from the STABILITY study randomized clinical trial. Am J Sports Med. 2020;48(2):285–297. 31940222 10.1177/0363546519896333

[13] Hecker A , Egli RJ , Liechti EF , Leibold CS , Klenke FM . Multiplanar reformation improves identification of the anterolateral ligament with MRI of the knee. Sci Rep. 2021;11(1):13216. 34168252 10.1038/s41598-021-92707-w PMC8225870

[14] Helito CP , Demange MK , Helito PVP , Costa HP , Bonadio MB , Pecora JR , et al. Evaluation of the anterolateral ligament of the knee by means of magnetic resonance examination. Revista Brasileira de Ortopedia (English Edition). 2015;50(2):214–219. 10.1016/j.rboe.2015.03.009 PMC4519625 26229919

[15] Helito CP , Helito PVP , Bonadio MB , Pécora JR , Bordalo‐Rodrigues M , Camanho GL , et al. Correlation of magnetic resonance imaging with knee anterolateral ligament anatomy. Orthop J Sports Med. 2015;3(12):2325967115621024. 26779553 10.1177/2325967115621024 PMC4710116

[16] Helito CP , Helito PVP , Costa HP , Bordalo‐Rodrigues M , Pecora JR , Camanho GL , et al. MRI evaluation of the anterolateral ligament of the knee: assessment in routine 1.5‐T scans. Skeletal Radiol. 2014;43(10):1421–1427. 25085699 10.1007/s00256-014-1966-7

[17] Helito CP , Helito PVP , Leão RV , Louza ICF , Bordalo‐Rodrigues M , Cerri GG . Magnetic resonance imaging assessment of the normal knee anterolateral ligament in children and adolescents. Skeletal Radiol. 2018;47(9):1263–1268. 29627859 10.1007/s00256-018-2933-5

[18] Henry BM , Tomaszewski KA , Ramakrishnan PK , Roy J , Vikse J , Loukas M , et al. Development of the anatomical quality assessment (AQUA) tool for the quality assessment of anatomical studies included in meta‐analyses and systematic reviews. Clin Anat. 2017;30(1):6–13. 27718281 10.1002/ca.22799

[19] Huang T , He X , Zhang L , Li C , Yang Y , Zhang J , et al. What is the anatomic footprint of the anterolateral ligament of the knee? A race‐ and sex‐based MRI analysis. Clin Orthop Relat Res. 2025;483(11):2147–2158. 40335065 10.1097/CORR.0000000000003519 PMC12517939

[20] Kang J‐H , Moon S‐G , Lee D‐W . Magnetic resonance imaging features of anterolateral ligament in young adults without anterior cruciate ligament injury: preliminary evaluation. Diagnostics. 2024;14(12):1226. 38928641 10.3390/diagnostics14121226 PMC11202545

[21] Kennedy MI , Claes S , Fuso FAF , Williams BT , Goldsmith MT , Turnbull TL , et al. The anterolateral ligament. Am J Sports Med. 2015;43(7):1606–1615. 25888590 10.1177/0363546515578253

[22] Kızılgöz V , Sivrioğlu AK , Aydın H , Çetin T , Ulusoy GR . Assessment of the anterolateral ligament of the knee by 1.5 T magnetic resonance imaging. J Int Med Res. 2018;46(4):1486–1495. 29350081 10.1177/0300060517740032 PMC6091844

[23] Klontzas ME , Maris TG , Zibis AH , Karantanas AH . Normal magnetic resonance imaging anatomy of the anterolateral knee ligament with a T2/T1‐weighted 3‐dimensional sequence: a feasibility study. Can Assoc Radiol J. 2016;67(1):52–59. 26702759 10.1016/j.carj.2015.08.004

[24] Kosy JD , Mandalia VI , Anaspure R . Characterization of the anatomy of the anterolateral ligament of the knee using magnetic resonance imaging. Skeletal Radiol. 2015;44(11):1647–1653. 26205762 10.1007/s00256-015-2218-1

[25] Kosy JD , Schranz PJ , Patel A , Anaspure R , Mandalia VI . The magnetic resonance imaging appearance of the anterolateral ligament of the knee in association with anterior cruciate rupture. Skeletal Radiol. 2017;46(9):1193–1200. 28432395 10.1007/s00256-017-2657-y

[26] Liebensteiner MC , Henninger B , Kittl C , Attal R , Giesinger JM , Kranewitter C . The anterolateral ligament and the deep structures of the iliotibial tract: MRI visibility in the paediatric patient. Injury. 2019;50(2):602–606. 30391071 10.1016/j.injury.2018.10.040

[27] Macchi V , Porzionato A , Morra A , Stecco C , Tortorella C , Menegolo M , et al. The anterolateral ligament of the knee: a radiologic and histotopographic study. Surg Radiol Anat. 2016;38(3):341–348. 26476833 10.1007/s00276-015-1566-9

[28] Marshall T , Oak SR , Subhas N , Polster J , Winalski C , Spindler KP . Can the anterolateral ligament be reliably identified in anterior cruciate ligament–intact and anterior cruciate ligament–injured knees on 3‐T magnetic resonance imaging? Orthop J Sports Med. 2018;6(9):2325967118796452. 30263895 10.1177/2325967118796452 PMC6153540

[29] Muramatsu K , Saithna A , Watanabe H , Sasaki K , Yokosawa K , Hachiya Y , et al. Three‐dimensional magnetic resonance imaging of the anterolateral ligament of the knee: an evaluation of intact and anterior cruciate ligament–deficient knees from the Scientific Anterior Cruciate Ligament Network International (SANTI) Study Group. Arthroscopy. 2018;34(7):2207–2217. 29730221 10.1016/j.arthro.2018.02.014

[30] Nasu H , Nimura A , Yamaguchi K , Akita K . Morphology of the anterolateral ligament: a complex of fibrous tissues spread to the anterolateral aspect of the knee joint. Anat Sci Int. 2020;95(4):470–477. 32347456 10.1007/s12565-020-00543-1 PMC7381439

[31] Olewnik Ł , Gonera B , Kurtys K , Podgórski M , Polguj M , Sibiński M , et al. The anterolateral ligament of the knee: a proposed classification system. Clin Anat. 2018;31(7):966–973. 30144325 10.1002/ca.23267

[32] Page MJ , McKenzie JE , Bossuyt PM , Boutron I , Hoffmann TC , Mulrow CD , et al. The PRISMA 2020 statement: an updated guideline for reporting systematic reviews. BMJ. 2021;372:n71. 33782057 10.1136/bmj.n71 PMC8005924

[33] Porrino J , Maloney E , Richardson M , Mulcahy H , Ha A , Chew FS . The anterolateral ligament of the knee: MRI appearance, association with the segond fracture, and historical perspective. Am J Roentgenol. 2015;204(2):367–373. 25615760 10.2214/AJR.14.12693

[34] Saithna A , Geeslin AG , Sonnery‐Cottet B . Lateral extra‐articular procedures with anterior cruciate ligament reconstruction: international consensus. Arthroscopy. 2025;41(9):3300–3302. 40975595 10.1016/j.arthro.2025.06.011

[35] Schwarzer G , Rücker G , Semaca C . *LFK* index does not reliably detect small‐study effects in meta‐analysis: a simulation study. Res Synth Methods. 2024;15(4):603–615. 38467140 10.1002/jrsm.1714

[36] Song Y , Yang J‐H , Choi WR , Lee JK . Magnetic resonance imaging‐based prevalence of anterolateral ligament abnormalities and associated injuries in knees with acute anterior cruciate ligament injury. J Knee Surg. 2019;32(09):866–871. 30189439 10.1055/s-0038-1669449

[37] Sonnery‐Cottet B , Carrozzo A , Saithna A , Monaco E , Vieira TD , Musahl V , et al. Indications for lateral extra‐articular procedures in the anterior cruciate ligament–reconstructed knee: Part I of an international consensus statement. Arthroscopy. 2025;41(9):3303–3312. 40544926 10.1016/j.arthro.2025.06.012

[38] Sonnery‐Cottet B , Vieira TD , Ouanezar H . Anterolateral ligament of the knee: diagnosis, indications, technique, outcomes. Arthroscopy. 2019;35(2):302–303. 30712610 10.1016/j.arthro.2018.08.019

[39] Taneja AK , Miranda FC , Braga CAP , Gill CM , Hartmann LGC , Santos DCB , et al. MRI features of the anterolateral ligament of the knee. Skeletal Radiol. 2015;44(3):403–410. 25427785 10.1007/s00256-014-2052-x

[40] Totlis T , Tishukov M , Piagkou M , Vasiliadis AV , Tsiouris C , Domashenko P , et al. The anterolateral ligament of the knee is a nonisometric thin ligament with high prevalence and almost constant Attachment to the lateral meniscus: a systematic review with meta‐analysis. Arthroscopy. 2024;40(4):1288–1299. 37832743 10.1016/j.arthro.2023.09.031

[41] Veizi E , Oliver‐Welsh L , Koutserimpas C , Imat E , Getgood A . Extra‐articular tenodesis for ACL reconstruction: who needs it and is there a superior technique? Curr Rev Musculoskelet Med. 2026;19(1):31. 41922887 10.1007/s12178-026-10028-9 PMC13043991

[42] Vincent JP , Magnussen RA , Gezmez F , Uguen A , Jacobi M , Weppe F , et al. The anterolateral ligament of the human knee: an anatomic and histologic study. Knee Surg Sports Traumatol Arthrosc. 2012;20(1):147–152. 21717216 10.1007/s00167-011-1580-3

[43] Wodicka R , Jose J , Baraga MG , Kaplan LD , Lesniak BP . MRI evaluation of the anterolateral ligament of the knee in the setting of ACL rupture. Orthop J Sports Med. 2014;2:2325967114S00042.

