# Supplementary material for: Magnetic resonance imaging‐based identification of the knee anterolateral ligament: A systematic review with meta‐analysis
Source: J Exp Orthop. 2026 Jun 26;13(3):e70816. doi: 10.1002/jeo2.70816 (PMC13307614; doi:10.1002/jeo2.70816)
Supplement: Supplementary file 1 — Supporting information file 1. [file JEO2-13-e70816-s001.docx]

**Supplementary Materials**

**
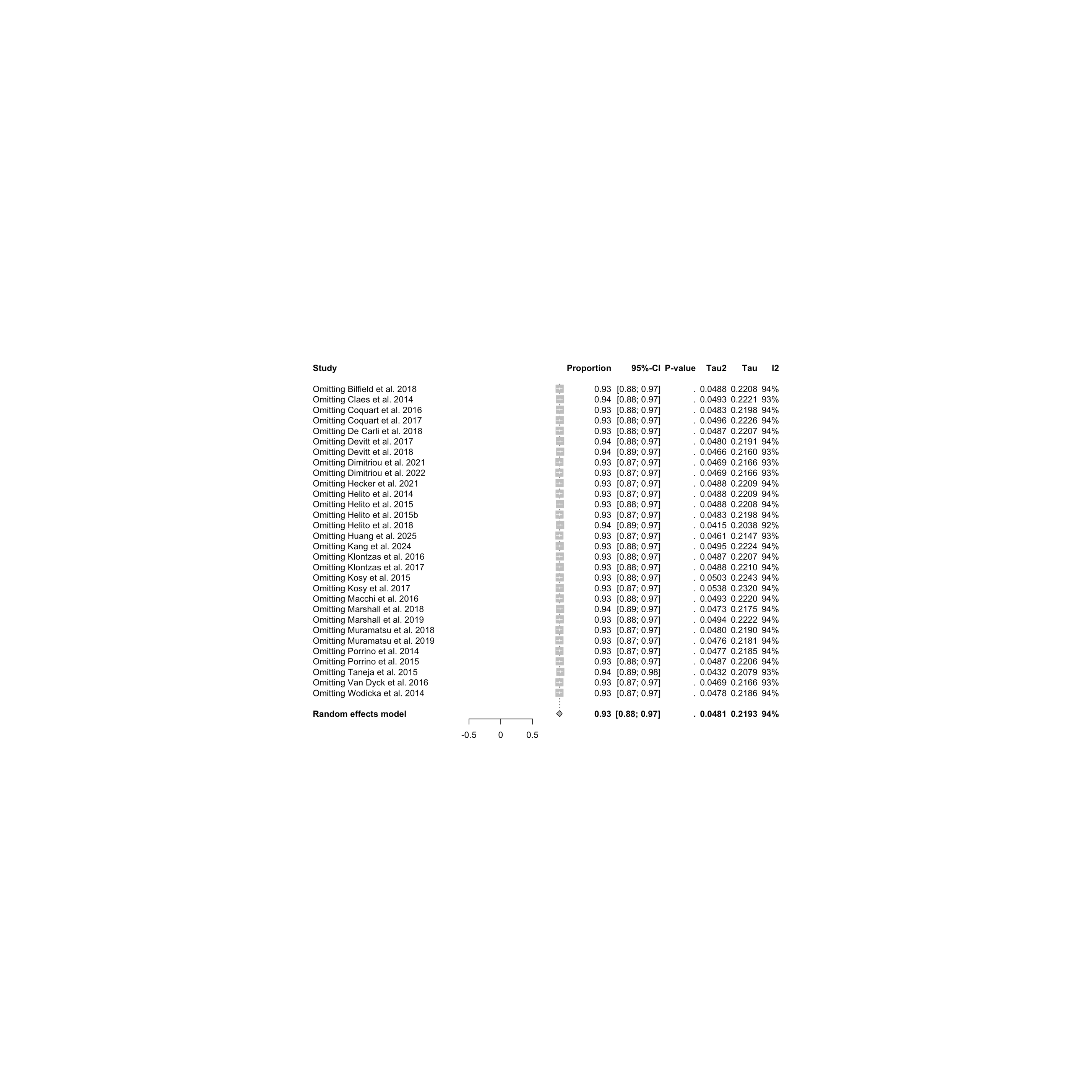
**

*Supplementary Figure 1.* Forest plot for the leave-one-out analysis of the pooled prevalence estimate of the anterolateral ligament visualization.


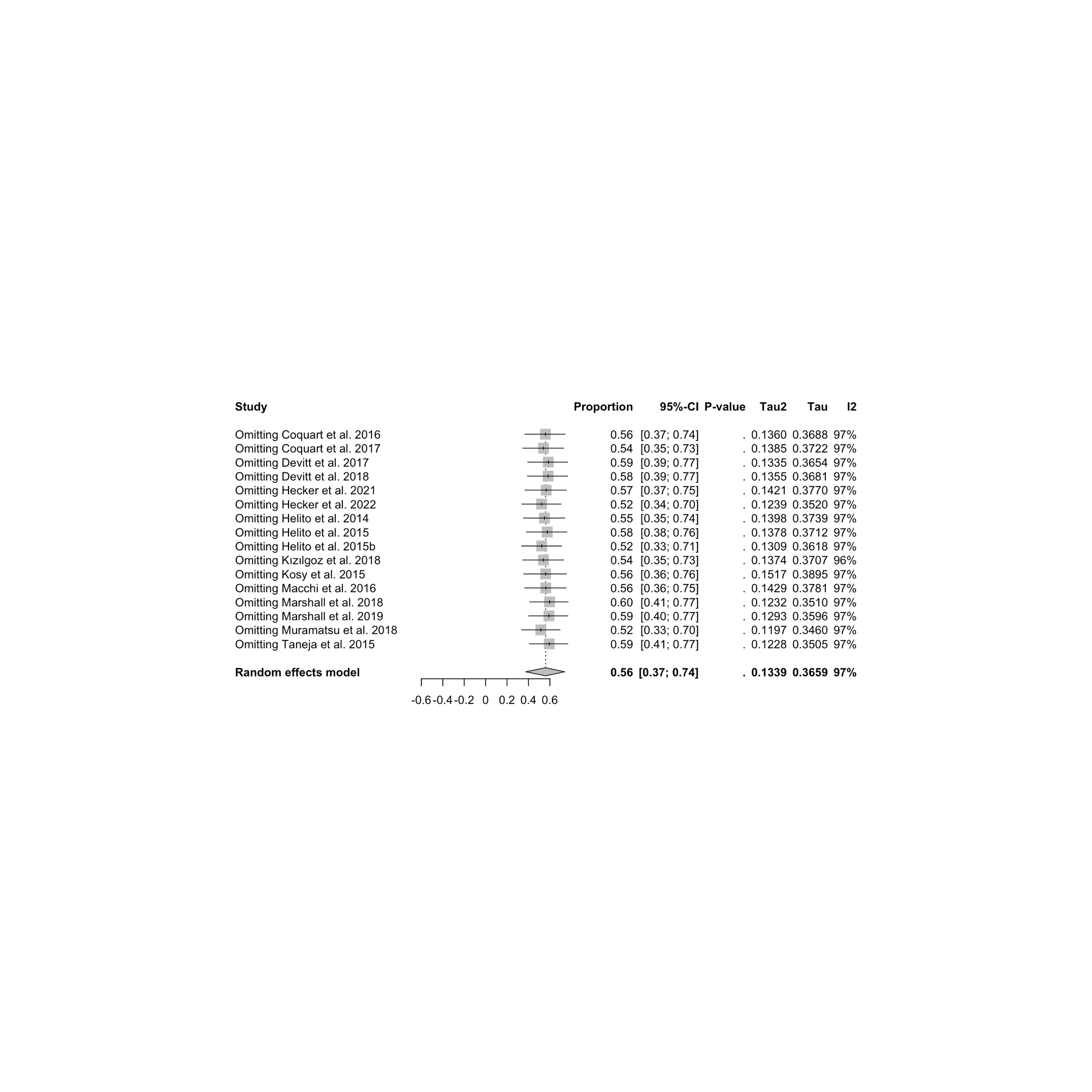


*Supplementary Figure 2.* Forest plot for the leave-one-out analysis of the pooled prevalence estimate of the anterolateral ligament complete visualization.


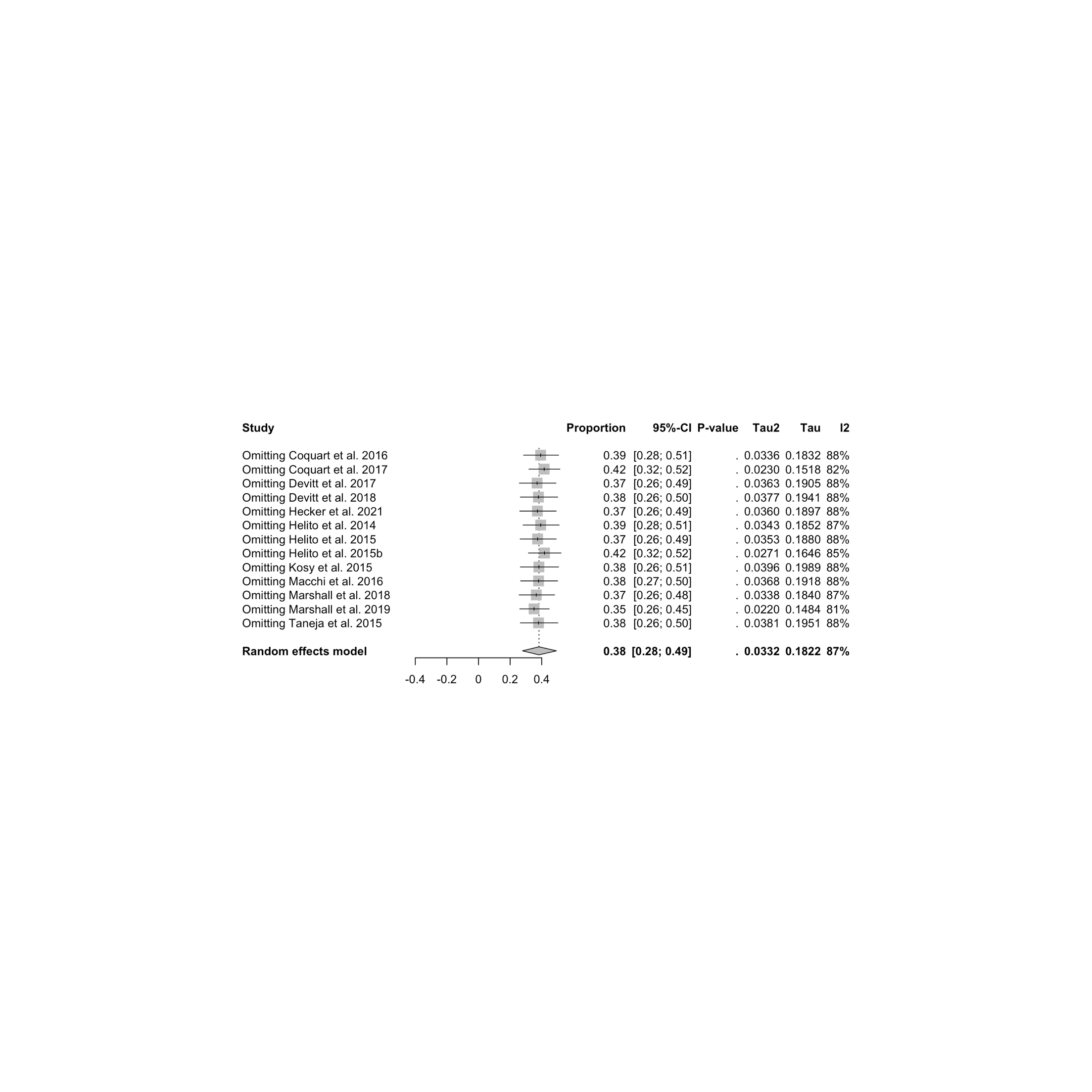


*Supplementary Figure 3.* Forest plot for the leave-one-out analysis of the pooled prevalence estimate of the anterolateral ligament partial visualization.


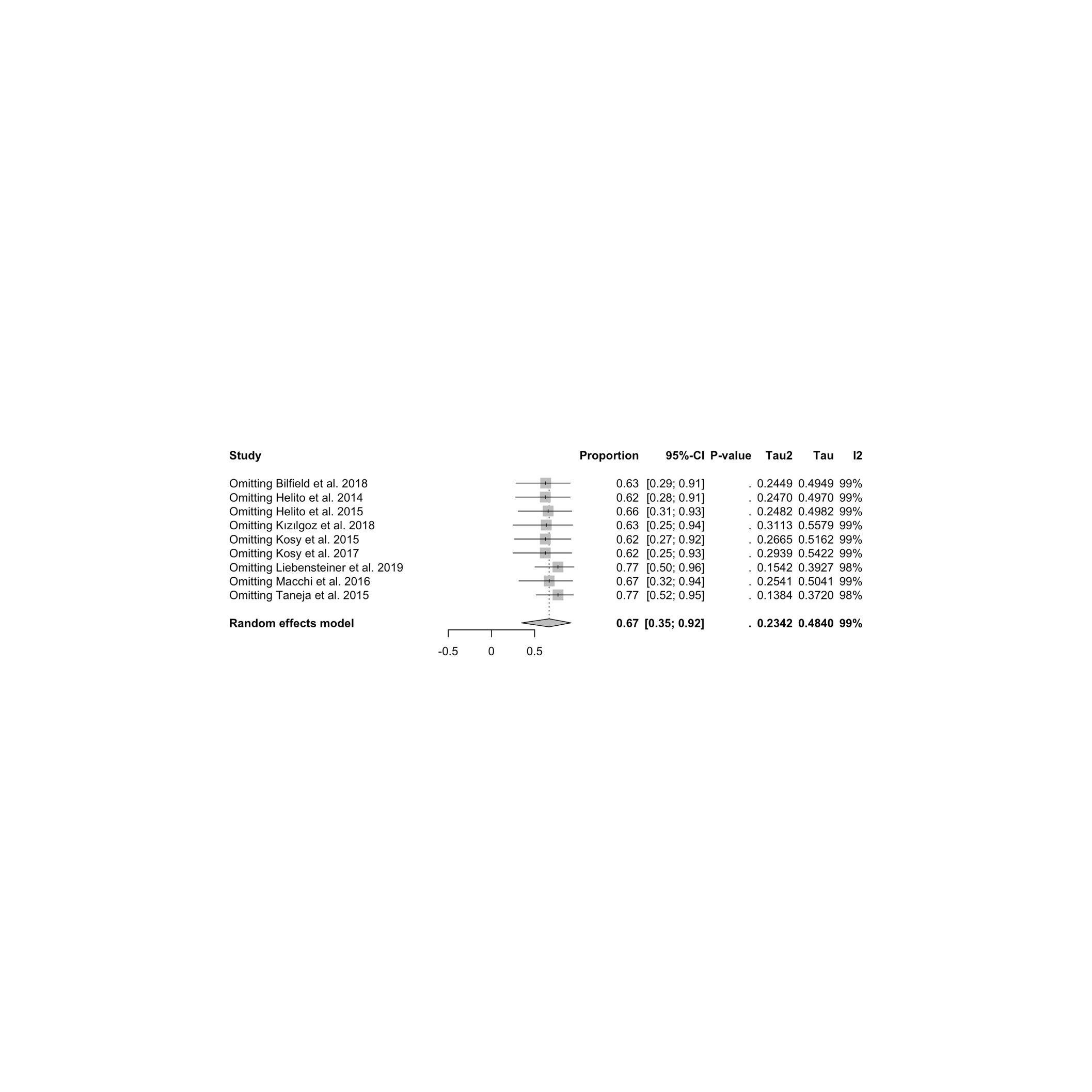


*Supplementary Figure 4.* Forest plot for the leave-one-out analysis of the pooled prevalence estimate of the anterolateral ligament meniscal part visualization.


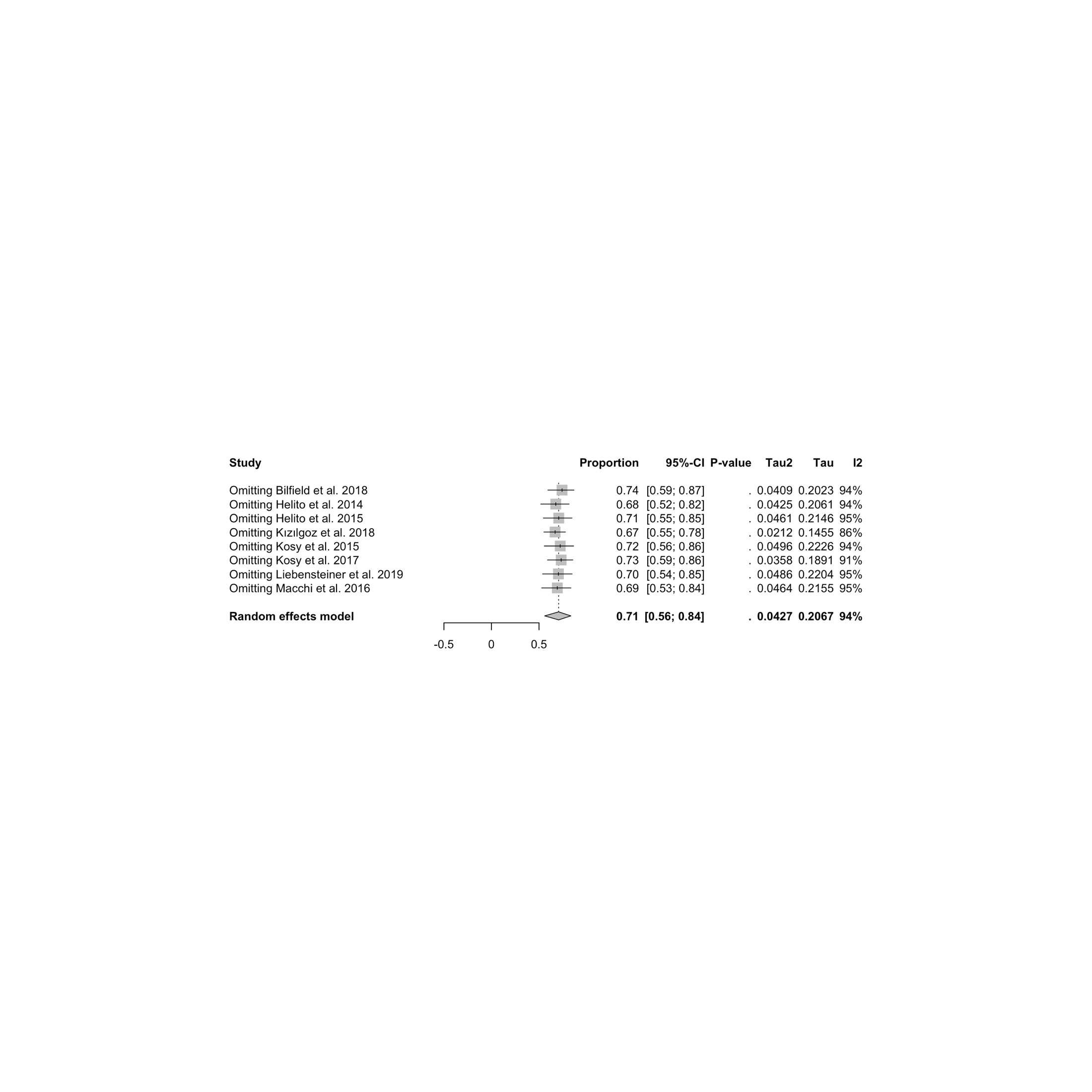


*Supplementary Figure 5.* Forest plot for the leave-one-out analysis of the pooled prevalence estimate of the anterolateral ligament femoral part visualization.


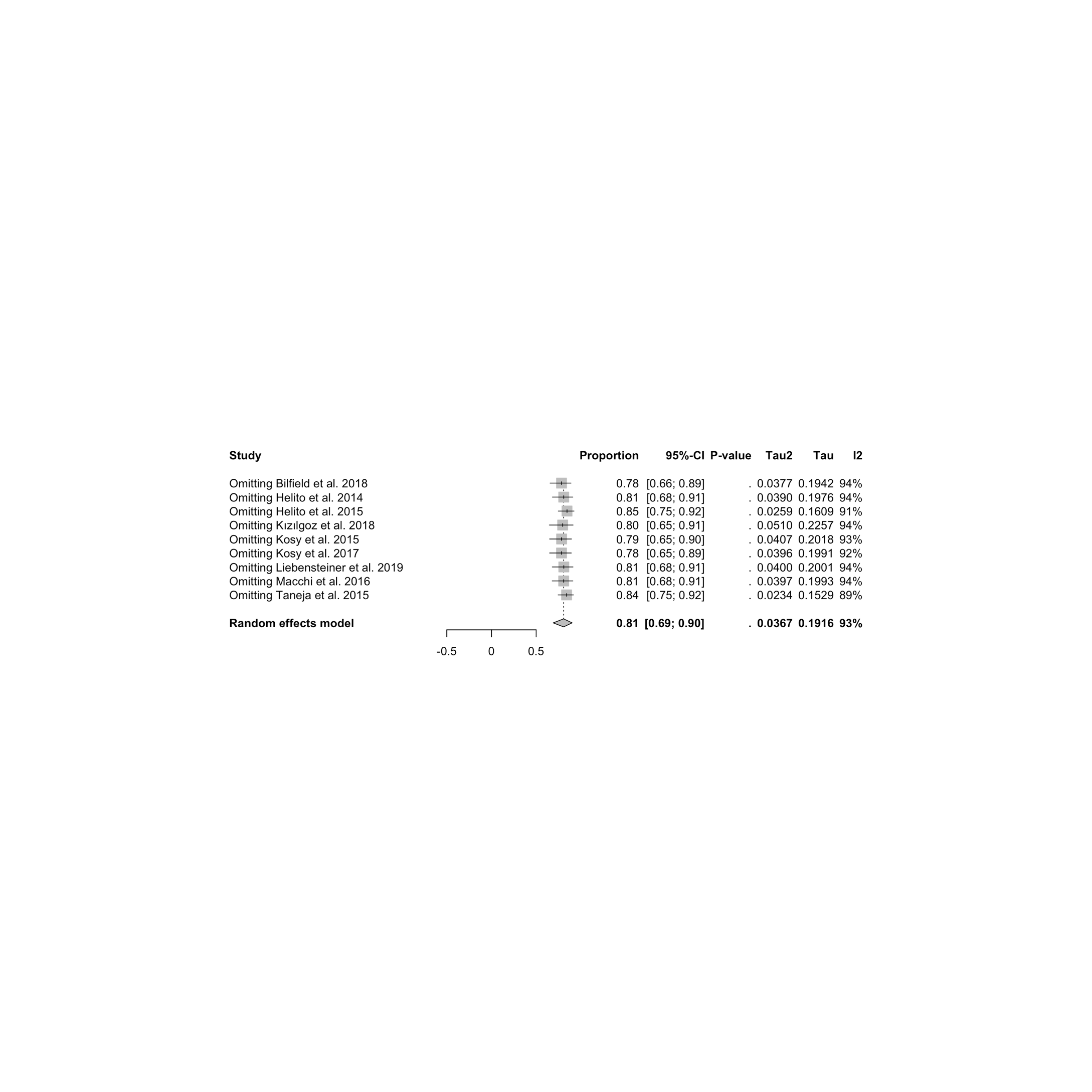


*Supplementary Figure 6.* Forest plot for the leave-one-out analysis of the pooled prevalence estimate of the anterolateral ligament tibial part visualization.
